# First report on the molecular prevalence and associated risk factors of *Eimeria* spp. in dairy cattle in Khon Kaen, Thailand

**DOI:** 10.14202/vetworld.2023.1489-1495

**Published:** 2023-07-19

**Authors:** Bamphen Keomoungkhoun, I Putu Gede Yudhi Arjentinia, Somboon Sangmaneedet, Weerapol Taweenan

**Affiliations:** Division of Pathobiology, Faculty of Veterinary Medicine, Khon Kaen University, Khon Kaen 40002, Thailand

**Keywords:** bovine coccidiosis, dairy cattle, *Eimeria* spp, molecular prevalence, multiplex polymerase chain reaction, Thailand

## Abstract

**Background and Aim:**

Bovine coccidiosis, caused by the protozoa *Eimeria*, is an important parasitic cattle disease that affects animal health and has economic impact worldwide. This study was conducted to report the first molecular prevalence and genetic diversity of *Eimeria* spp. in dairy cattle in Khon Kaen province, Thailand, and to identify the risk factors associated with *Eimeria* spp. infection.

**Materials and Methods:**

From July 2020 to October 2021, 296 fecal samples were collected from dairy cattle divided into three age groups, including <3-month-old calves, 3-month-old to 1-year-old calves, and >1-year-old cattle. *Eimeria* spp. were identified by multiplex polymerase chain reaction (PCR) amplifying 18S RNA gene and confirmed by DNA sequencing. Information regarding all associated risk factors was collected using questionnaires and analyzed using logistic regression tests in the Statistical Package for the Social Sciences program.

**Results:**

Polymerase chain reaction results showed that 104 (35.13%) of 296 samples were positive for *Eimeria* spp. The <3-month-old calves (46.51%) had the highest infection rate. Moreover, multiplex PCR identified five species of *Eimeria*, namely, *Eimeria* *bovis* (32.69%), *Eimeria* *zuernii* (18.26%), *Eimeria* *alabamensis* (5.76%), *Eimeria ellipsoidalis* (3.84%), and *Eimeria* *cylindrica* (2.88%). An association was observed between risk factors and *Eimeria* spp. incidence (p < 0.05). DNA sequencing revealed the similarity of each *Eimeria* spp. with 91%–100% nucleotide identity. Phylogenetic tree analysis demonstrated the close relationships of clusters of *E. bovis* and *E. zuernii*, *E. ellipsoidalis*, and *E. cylindrica* and another cluster of *E. alabamensis*.

**Conclusion:**

The results confirm that *Eimeria* spp. are commonly found in dairy cattle, especially calves. The molecular test could be powerful for species identification. This study also provides epidemiological information for developing future strategies to control bovine coccidiosis.

## Introduction

Bovine coccidiosis caused by the protozoa *Eimeria*, is an important parasitic disease of cattle that affects animal health and exerts an economic impact worldwide. Although the economic impact of bovine coccidiosis is seldom investigated, there have been a number of reports of productivity losses and value of control and prevention. For instance, USD 23.78 million was reported in Mexico, USD 62 million in the United States [1] and USD 3.8 million in Canada [2]. In the neighboring countries of Thailand, the prevalence of *Eimeria* spp. in cattle was reported as 47% in China [3], 53% in Indonesia [4], and 56%–90% in Malaysia [5, 6]. In general, the clinical signs of coccidiosis include weakness, poor appetite, weight loss, fever, anemia, and diarrhea [7]. Severe diarrhea was found in infected calves aged <1 year [8]. According to previous studies by Yusof and Isa [5], Yusof [6], the prevalence of *Eimeria* spp. reaches up to 100% in calves [9] and 3%–60% in adults. Furthermore, a high prevalence of *Eimeria* infection was recorded in calves compared with adults. Rainy seasons and poor hygienic conditions are also considered as risk factors associated with *Eimeria* infection [5, 7, 8].

At present, 13 species of *Eimeria* are reported in cattle [7]. *Eimeria bovis* and *Eimeria zuernii* are confirmed as the predominant species causing high pathogenesis, particularly clinical diarrhea, in calves [8]. Although the microscopic examination is the gold standard for detecting this parasite, the diagnosis becomes confusing due to the highly close similarity of the morphological oocysts of each *Eimeria* species. Therefore, molecular techniques, especially polymerase chain reaction (PCR), have been developed to detect parasitic DNA [10]. In particular, multiplex PCR is a technique widely used to detect common *Eimeria* spp. in cattle, such as *Eimeria alabamensis*, *Eimeria* *auburnensis*, *E. bovis*, *Eimeria cylindrica*, *Eimeria ellipsoidalis*, and *E. zuernii*. This PCR technique is extremely useful for accurately diagnosing *Eimeria* spp. in cattle [11].

Studies on the molecular prevalence and its risk factor association are extremely important; however, there has yet to be no research on identifying *Eimeria* spp. in Khon Kaen province, Thailand. In addition, a rapid and accurate diagnosis would be essential for developing control programs and reducing the amount of unnecessary antiparasitic drug treatment.

Therefore, this study was conducted to report the first molecular prevalence and genetic diversity of *Eimeria* spp. in dairy cattle in Khon Kaen province, Thailand. Moreover, the risk factors associated with *Eimeria* spp. infection were identified.

## Materials and Methods

### Ethical approval

This study was approved by the Institutional Animal Care and Use Committee of Khon Kaen University, recorded no. IACUC-KKU-126_64 and reference no. 660201.2.11/646 (121).

### Study period and location

**Figure-1:**
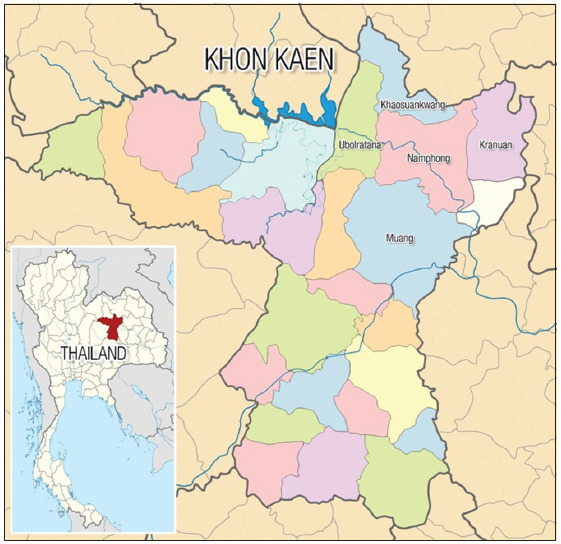
Location of the study area (source: https://fr.m.wikipedia.org/wiki/Fichier: Thailand_Khon_Kaen_location_map.svg).

The study was conducted from July 2020 to October 2021. This study was conducted on 43 smallholder dairy cattle farms in five districts (Muang, Namphong, Kranuan, Khaosuankwang, and Ubolratana district) of Khon Kaen Province, Northeast Thailand (Figure-1). Khon Kaen is located at 16°26’N and 102°50’E with an altitude of 150–200 m above sea level. The annual average temperature is approximately 26.9°C, with the minimum, and maximum being 22.3°C and 32.8°C, respectively. The average annual rainfall is 1234.7 mm [12].

### Study population, and fecal sample collection

The sample size for this study was based on a 48% prevalence of *Eimeria* spp. among dairy calves in Khon Kaen province [13], with an allowable error of 6%.

A total of 296 fecal samples were collected from dairy cattle divided into three age groups, namely, <3-month-old calves, 3-month-old to 1-year-old calves, and >1-year-old cattle. The fecal samples were directly collected from the rectum of cattle. All samples were stored in the refrigerator at 4°C in the laboratory of the Faculty of Veterinary Medicine, Khon Kaen University, until examination.

### Questionnaires

Questionnaires were designed to identify the risk factors for *Eimeria* spp. infection in dairy cattle. Questions were related to general information about owners, including gender, age, job, education, and address. Information regarding individual cattle, including sex, age, and vaccination status, was also added to the questionnaire. The questionnaire also included the potential risk factors such as the number of cattle in the herd, type of feeder, type of water trough, and type of house floor. The frequency of feeders, water troughs, and house floor cleaning were also added to the questionnaire.

### DNA extraction

Genomic DNA was extracted from all fecal samples using the GF1 soil sample DNA extraction kit (Vivantis, Malaysia) according to the manufacturer’s instructions. Briefly, 250 mg of fecal sample and 1 mL of buffer SL1 were added into a 2-mL microcentrifuge tube containing 0.5 g of glass beads. The mixture was vortexed for 5 min, incubated at 72°C for 10 min, and again vortexed twice under incubation. Then, the procedure was continued by following the manufacturer’s instruction. Finally, the DNA was eluted with 100 μL of elution buffer, left to stand for 2 min, centrifuged at 10,000× *g* for 1 min, transferred to a new 1.5-mL microcentrifuge tube, and stored at −20°C until PCR analysis.

### Amplification of first internal transcribed spacer (ITS-1) region

Forward and reverse primers of *Eimeria* genus (Table-1) [14] were used to amplify DNA that holds on the ITS-1 of the *18S rRNA* gene. Polymerase chain reaction was performed using a 20-μL reaction mixture consisting of 9 μL of water, 10 μL of master mix, 0.25 μL of each primer (total 0.5 μL), and 0.5 μL of DNA template. The first PCR step was performed with an initial denaturation step at 95°C for 5 min, followed by 30 cycles of 95°C for 30 s, 58°C for 30 s, 72°C for 45 s, and an elongation step at 72°C for 5 min [14]. All PCR product sizes were analyzed using 1% agarose gel electrophoresis, and products with 348–546 bp were later processed for species identification by multiplex PCR.

**Table-1:** Primers for multiplex PCR [14].

Species	Expected product size (bp)	Forward primer	Reverse primer
Common genus	348–546	5’- GCAAAAGTCGTAACACGGTTTCCG-3’	5’- CTGCAATTCACAATGCGTATCGC-3’
*Eimeria alabamensis*	184	5’–CATTCACACATTGTTCTTTCAG–3’	5’–GCTTCCAAACTAATGTTCTG–3’
*Eimeria auburnensis*	295	5’–TAAATTGGTGCGATGAGGGA–3’	5’–GCAATGAGAGAAAGATTTAATA–3’
*Eimeria bovis*	238	5’–TCATAAAACATCACCTCCAA–3’	5’–ATAATTGCGATAAGGGAGACA–3’
*Eimeria cylindrica*	304	5’–GACATTTAAAAAACCGATTGGT–3’	5’–GGCTGCAATAAGATAGACATA–3’
*Eimeria ellipsoidalis*	148	5’–CAACGTTTTTCCTTTTCCTATCA–3’	5’–ACTGCGATGAGAGAGAGCG–3’
*Eimeria zuernii*	344	5’–AACATGTTTCTACCCACTAC–3’	5’–CGATAAGGAGGAGGACAAC–3’

PCR=Polymerase chain reaction

### Multiplex PCR

Forward and reverse primers (Table-1) were used to amplify the DNA of each *Eimeria* spp. The reaction was run in a 20-μL mixture consisting of 6.5 μL of water, 10 μL of master mix, 0.25 μL of each forward primer (total 1.5 μL), 0.25 μL of each reverse primer (total 1.5 μL), and 0.5 μL of the amplicon from the first round PCR as the DNA template. The PCR procedure was performed similarly to that of the first reaction. Then, 5 μL of each PCR product was identified using 1% agarose gel electrophoresis with 100 bp of the ladder in 30 min [14]. The gel was observed visually by Visafe Red Gel Stain (Vivantis) under ultra-violet transillumination.

### DNA sequence analysis

The PCR products of positive samples were sequenced using BTSeq™ (Barcode-Tagged Sequencing; CELEMICS, Seoul, Korea). Each sequence was compared with *Eimeria* spp. references in the GenBank database using BLAST. The phylogenetic tree was constructed using the neighbor-joining method with 1000 replicates for bootstrap analysis using the MEGA 11 (https://www.megasoftware.net/).

### Statistical analysis

The relationship between associated risk factors and prevalence was analyzed using logistic regression in the Statistical Package for the Social Sciences program version 19 (IBM Corp., NY, USA). p < 0.05 was considered for statistical significance.

## Results

### Prevalence of *Eimeria* spp

**Figure-2:**
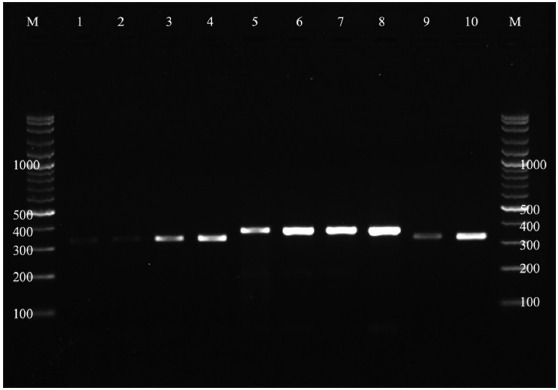
Agarose gel electrophoresis of *Eimeria* genus positive samples. M = Marker (100–2000 bp), Lane 1–10 = positive samples at 348–546 bp of polymerase chain reaction product.

Of the 296 fecal samples, 104 (35.13%) were positive for *Eimeria* spp. according to conventional PCR (Table-2). All samples were investigated for *Eimeria* based on the 348–546 -bp size on agarose gel electrophoresis (Figure-2).

**Table-2:** Prevalence of *Eimeria* spp. in dairy cattle in five districts of Khon Kaen.

No.	Area	% prevalence (no. of positive/no. of samples)	95% CI
1	Muang	38.23 (26/68)	32.70–43.76
2	Namphong	65.00 (13/20)	59.57–70.43
3	Ubolratana	26.49 (31/117)	21.47–31.51
4	Kranaun	44.44 (20/45)	38.78–50.10
5	Khaosaunkwang	30.43 (14/46)	25.19–35.67
Total		35.13 (104/296)	29.70–40.56

CI=Confidence interval

### *Eimeria* spp. identification

**Figure-3:**
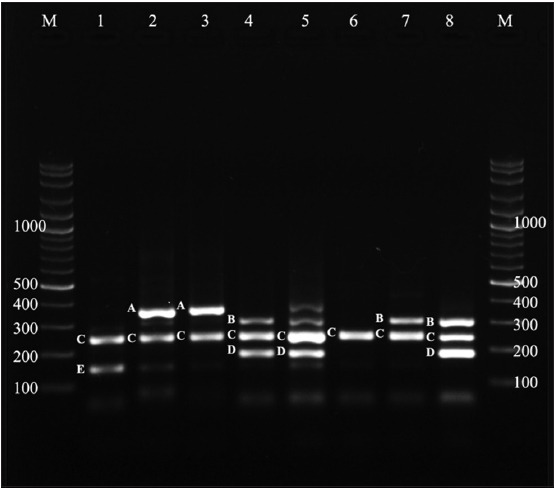
Agarose gel electrophoresis for multiplex polymerase chain reaction product analysis of *Eimeria* species positive samples. M = Marker (1500–100 bp), Lane = 1 - 8 positive samples at different base pair: A = *Eimeria zuernii* (350 bp); B = *Eimeria cylindrica* (304 bp); C = *Eimeria bovis* (238 bp); D = *Eimeria alabamensis* (184 bp); E = *Eimeria ellipsoidalis* (148 bp).

Results of the multiplex PCR showed that 36.54% (38/104) of the fecal samples were positive for mixed infection, among which five species of *Eimeria* were identified. The predominant species was *E. bovis* (61.53%), followed by *E. zuernii* (38.46%), *E. ellipsoidalis* (18.26%), *E. alabamensis* (15.38%), and *E. cylindrica* (8.65%) (Table-3). Specific primers of each *Eimeria* species were used to examine positive samples by multiplex PCR (Figure-3).

**Table-3:** *Eimeria* species identification by multiplex PCR.

Species	% prevalence (no. of positive/no. of samples)
Single infection	65.26 (66/104)
*E. bovis*	32.69 (34/104)
*E. zuernii*	18.26 (19/104)
*E. alabamensis*	5.76 (6/104)
*E. ellipsoidalis*	3.84 (4/104)
*E. cyllindrica*	2.88 (3/104)
Mixed infection	36.53 (38/104)
*E. bovis + E. zuernii*	9.61 (10/104)
*E. bovis + E. ellipsoidalis*	9.61 (10/104)
*E. zuernii + E. cylindrica*	4.80 (5/104)
*E. bovis + E. alabamensis*	3.84 (4/104)
*E. zuernii + E. alabamensis*	0.96 (1/104)
*E. zuernii + E. ellipsoidalis*	0.96 (1/104)
*E. bovis + E. alabamensis + E. ellipsoidalis*	2.88 (3/104)
*E. bovis + E. zuernii +* *E. alabamensis*	1.92 (2/104)
*E. zuernii + E. alabamensis + E. cylindrica*	0.96 (1/104)
*E. bovis + E. ellipsoidalis + E. zuernii*	0.96 (1/104)

PCR=Polymerase chain reaction, *E. bovis*=*Eimeria bovis, E. zuernii*=*Eimeria zuernii, E. alabamensis*=*Eimeria alabamensis, E. ellipsoidalis*=*Eimeria ellipsoidalis,* *E. cyllindrica*=*Eimeria cylindrica*

### Prevalence of *Eimeria* spp. and associated risk factors

According to the statistical analysis, the prevalence of *Eimeria* spp. in dairy cattle was significantly associated with the age of cattle (p < 0.05) (Table-4). Calves (<3 months old) showed a high risk for *Eimeria* infection, with the odds ratio (OR) being 2.14 compared with adults (>1 year old). Older calves (3 months old to 1 year old) showed no association with *Eimeria* occurrence (p > 0.05) compared with adults (>1 year old), with the OR being 1.48.

**Table-4:** Associated risk factors and *Eimeria* prevalence.

Factors	% prevalence (no. of positive/no. of samples)	Univariable analysis		Multivariable analysis	
	
		Odds ratio (95% CI)	p-value	Adjusted odds ratio (95% CI)	p-value
Age					
>1 year old	28.80 (36/125)	1		1	
3 months–1 year old	37.50 (48/128)	1.48 (0.87–2.51)	0.14	0.71 (0.40–1.25)	0.24
<3 months old	46.51 (20/43)	2.14 (1.05–4.38)	0.04*	1.30 (0.63–2.71)	0.47
Feeder box cleaning					
Every day	30.33 (64/211)	1		1	
Once a week	47.05 (40/85)	2.04 (1.21–3.42)	0.009**	2.54 (1.42–4.50)	0.002**
Water trough type					
Moveable container	28.16 (40/142)	1		1	
Concrete tank	41.55 (64/154)	1.81 (1.11–2.94)	0.02*	1.97 (0.85–4.54)	0.11
House floor type					
Concrete	29.56 (55/186)	1		1	
Ground	44.54 (49/110)	1.86 (1.14–3.00)	0.01**	1.33 (0.69–2.55)	0.42
Water trough cleaning					
Once a week	31.79 (62/195)	1		1	
Longer than a week	41.58 (42/101)	1.52 (0.92–2.51)	0.09	1.19 (0.61–2.33)	0.59
House floor cleaning					
Once a month	34.68 (77/222)	1		1	
Longer than a month	36.48 (27/74)	1.08 (0.62–1.87)	0.88	0.80 (0.42–1.51)	0.50

* Statistically significant difference (p ≤ 0.05).

** Statistically significant difference (p ≤ 0.01). CI=Confidence interval

Furthermore, other risk factors, including feeder box cleaning, water trough type, and house floor type, were significantly associated with *Eimeria* prevalence (p < 0.05) (Table-4). Cattle reared in farms with the feeder box cleaning done once a week (OR = 2.04), cattle reared in farms using concrete tanks as drinking water containers (OR = 1.81), and cattle housed on the ground floor (OR = 1.86) showed a high associated risk for *Eimeria* spp. infection. However, no association was found between *Eimeria* prevalence and the frequency of water trough cleaning and the frequency of house floor cleaning (p > 0.05).

### Sequencing and phylogenetic tree analysis

**Figure-4:**
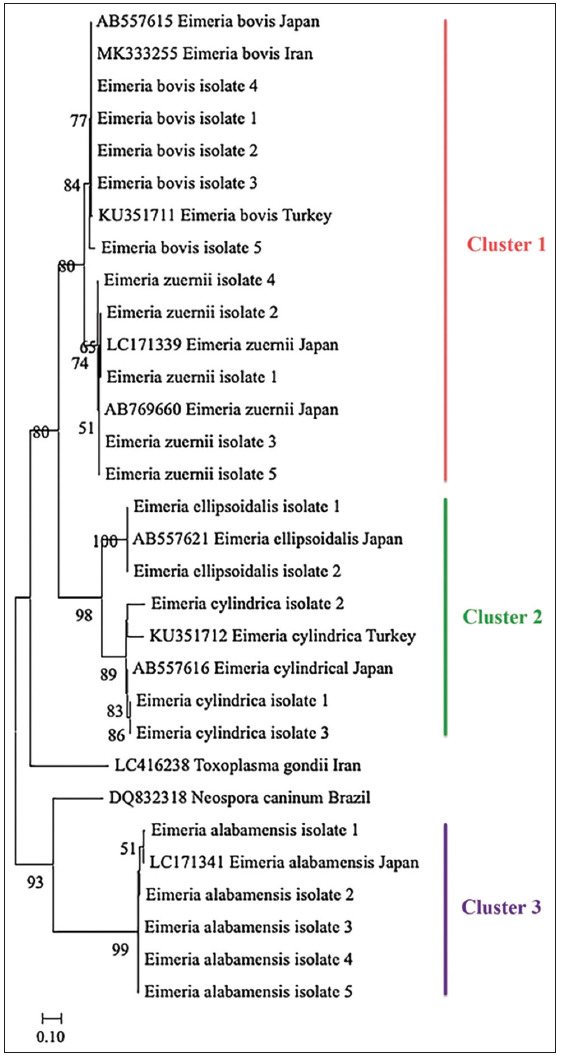
Phylogenetic tree of local *Eimeria* spp. isolates. The phylogenetic tree was constructed using the maximum likelihood method. Numbers indicate percentages (1000 replicates) in the MEGA 11.0 version.

The positive samples of five species were randomly selected for sequencing with the sequence IDs, namely *E. bovis* isolate 1–5, *E. zuernii* isolate 1–5, *E. alabamensis* isolate 1–5, *E. cylindrica* isolate 1–3, and *E. ellipsoidalis* isolate 1–2. The phylogenetic tree was constructed from the local *Eimeria* isolates based on *18S rRNA*, *ITS-1* gene sequence, and the reference *Eimeria* species from GenBank. Based on the alignment of the references from GenBank and the local *Eimeria* isolates from the present study using BLAST (https://blast.ncbi.nlm.nih.gov/Blast.cgi), the local *Eimeria* spp. isolates were found to be closely related to the *Eimeria* species from Japan, Turkey, and Iran, with 91.47%–100% nucleotide identity (Figure-4).

The phylogenetic tree revealed that the local *Eimeria* spp. isolates were closely related to each other and matched with the references *E. bovis* (KU351711, AB557615, MK333255), *E. zuernii* (AB769660, LC171339), *E. ellipsoidalis* (AB557621), *E. cylindrica* (KU351712, AB769616), and *E. alabamensis* (LC171341). According to the phylogenetic tree, there were three clusters: cluster 1 of *E. bovis* and *E. zuernii*, cluster 2 of *E. ellipsoidalis* and *E. cylindrica*, and cluster 3 of *E. alabamensis*. The phylogenetic tree outgroup *Toxoplasma gondii* (LC416238) demonstrated a similarity with the large group of *E. bovis* and *E. zuernii* and *E. ellipsoidalis* and *E. cylindrica*. Simultaneously, another outgroup, *Neospora caninum* (DQ832318), exhibited similarity to *E. alabamensis* sequences in the present study (Figure-4).

## Discussion

Bovine coccidiosis is commonly found in dairy cattle worldwide. The present study demonstrated that the overall prevalence of *Eimeria* spp. (35%) is lower than previously reported data (48%) by Keomoungkhoun and Taweenan [13] showing the prevalence of *Eimeria* spp. only in calves aged <6 months in Khon Kaen. Importantly, this pathogen has been primarily detected and causes significant protozoan infection in young animals, especially calves [9]. As the present study investigated dairy cattle with a wide age range from 1 week to 7 years, the overall prevalence of *Eimeria* is low. Furthermore, the current prevalence is lower than other reports in Malaysia (56%) [6], Indonesia (53%) [4], and China (47%) [3] because of various factors, including the age of animals, climate, management systems, and husbandry practices [14, 15].

The mixed infection found in the present study is consistent with previous findings by Dong *et al*. [3] and Cardim *et al*. [16]. The five *Eimeria* species detected in this study, namely, *E. bovis*, *E. zuernii*, *E. ellipsoidalis*, *E. alabamensis*, and *E. cylindrica*, were different from those of a previous study that reported two *Eimeria* spp. (*E. bovis* and *E. zuernii*) in Khon Kaen province, Thailand, examined using the flotation technique [13]. The PCR technique has been extensively used for *Eimeria* spp. identification because of its higher sensitivity than morphological examination under a light microscope [17]. However, *E. bovis* and *E. zuernii* were the predominant species found in this study, consistent with other studies conducted in Indonesia [4], China [3], Iraq [14], Iran [18], Brazil [16], Ethiopia [19], and Germany [8].

The risk factor analysis showed that age was significantly associated (p < 0.05) with *Eimeria* infection. Calves aged <3 months had a higher risk for infection than cattle aged >1 year. This finding is strongly consistent with several previous studies by Rehman *et al*. [2], Alemayehu *et al*. [19], Lopez-Osorio *et al*. [20] and Tamrat *et al*. [21] demonstrating that *Eimeria* infection caused serious problems in calves compared with adults. This is because adults possess higher protective immunity against *Eimeria* infection than calves. In contaminated environment, adults develop immunity when they repeat the intake of oocysts [22]. Moreover, *Eimeria* infection is considered a cause of economic problems in calves aged <3 months [9]. Nevertheless, this disagrees with another study reporting that there was no significant difference in *Eimeria* infection between calves aged <2 years and adults aged >2 years [23].

Furthermore, a significant correlation was observed between *Eimeria* incidence and house floor type (p < 0.05). Animals reared on the ground house floor had a higher risk for *Eimeria* infection than those reared on the concrete house floor. This finding is consistent with a previous report by Rehman *et al*. [2], which demonstrated a significant association between cattle reared on the noncement floor type and *Eimeria* infection because it is easier to clean the concrete floor than the ground floor. Moreover, cattle reared in farms with concrete tank water showed a higher risk for *Eimeria* infection than cattle reared in farms with pipe water because of a greater chance of *Eimeria* oocyst contamination in drinking water in the concrete tank than that in the pipe system. This is also consistent with a previous study by Ekawasti *et al*. [24], which demonstrated a higher prevalence of *Eimeria* infection in animals reared in farms with a low frequency of feeding equipment cleaning. Cattle fed with feeder boxes that were cleaned once a week showed a higher risk for *Eimeria* infection (p < 0.05) than those fed with feeder boxes that were cleaned daily. This finding is consistent other published investigations showing that calves reared with poor hygiene showed a higher risk of coccidiosis incidence than calves reared with good hygiene. Good hygiene could reduce the extent of *Eimeria* oocyst contamination on the housing floor, in feed, and in the water [21].

The phylogenetic tree analysis in this study indicated that the *Eimeria* isolates in Khon Kaen were closely related to the *Eimeria* references recorded on GenBank. Three clusters of *Eimeria* species, including cluster *E. alabamensis*, cluster *E. bovis* and *E. zuernii*, and cluster *E. cylindrica* and *E. ellipsoidalis*, were identified. These findings agree with a previous study by Asfaw and Ibrahim [23] and Heidari *et al*. [25], showing that *E. bovis* and *E. zuernii* were closely related in the same cluster. The results of the present study are also consistent and confirmed with a previous report by Malek and Kuraa [17] demonstrating that *E. bovis* and *E. zuernii* were closely related and not distant from *E. ellipsoidalis* and *E. cylindrica*.

## Conclusion

*Eimeria* spp. are commonly found in dairy cattle in Khon Kaen, especially in calves. *E. bovis* and *E. zuernii* are the most predominant species. Calves aged <3 months showed the highest risk for *Eimeria* infection. Feeder box cleaning (once a week), water trough type (concrete tank), and house floor (ground) were identified as the risk factors associated with *Eimeria* infection. The phylogenetic tree analysis revealed a close relationship between the clusters *E. zuernii* and *E. bovis*, *E. ellipsoidalis* and *E. cylindrica*, and *E. alabamensis*. These data could provide epidemiological information for developing future strategies to control bovine coccidiosis.

## Authors’ Contributions

BK: Data curation, investigation, formal analysis, and writing the original manuscript. BK and IPGYA: Sample collection, methodology, and resources. WT and SS: Resources, supervision, and validation. WT: Conceptualization, funding acquisition, project administration, review, and editing manuscript. All authors have read, reviewed, and approved the final manuscript.
